# Combined aromatherapy and music or sound for emotional distress: a systematic review of controlled studies published between 2019 and 2026

**DOI:** 10.3389/fpsyg.2026.1955987

**Published:** 2026-10-08

**Authors:** Haojian Zhai, Xinyue Chen, Dong Lv, Amy Jing-Wen Yin, Wenge Zhang, Yunkai Wang, Qiang Li, Zhixiong Lin

**Affiliations:** 1 Department of Psychiatry, Affiliated Hospital of Guangdong Medical University, Zhanjiang, Guangdong, China; 2 Neuromodulation Center, Dongguan Seventh People’s Hospital, Dongguan, Guangdong, China; 3 School of Rehabilitation Science, Shanghai University of Traditional Chinese Medicine, Shanghai, China; 4 College of Art and Design, Lingnan Normal University, Zhanjiang, Guangdong, China

**Keywords:** anxiety, aromatherapy, emotional distress, multisensory intervention, music therapy, systematic review

## Abstract

**Background:**

Emotional distress is common around clinical procedures, cancer supportive care, and health-professions training. Aromatherapy and music are easy to deliver, yet it remains unclear when combining them is worthwhile and whether the combination offers more than either component alone.

**Objective:**

To synthesize experimental studies of aromatherapy combined with music or sound published from January 2019 through September 7, 2026; identify the populations and settings in which benefits were most consistent; and separate benefit over inactive care from incremental benefit over a single sensory intervention.

**Methods:**

We searched PubMed, Embase, CINAHL, APA PsycINFO, Scopus, Web of Science Core Collection, CENTRAL, CNKI, and Dentistry & Oral Sciences Source from January 1, 2019, to September 7, 2026. Randomized trials and controlled nonrandomized or quasi-experimental studies were eligible when they included an identifiable arm combining aromatherapy with music or sound and reported emotional-distress or related physiological outcomes. Two reviewers independently screened records, extracted data, and assessed risk of bias. Clinical and methodological heterogeneity precluded meta-analysis; findings were synthesized narratively using SWiM guidance.

**Results:**

Twenty-four studies involving 2,530 participants from 12 countries or regions were included. Of these, 21 were randomized studies and three were controlled nonrandomized or quasi-experimental studies. The evidence covered procedural and perioperative care, dentistry, nursing education and occupational stress, and cancer or palliative care. Results were most consistent in pediatric dentistry, magnetic resonance imaging, nursing examinations or skills testing, and some repeated cancer-support programs. Two lithotripsy trials and the adult endodontic study did not show a clear benefit. Although combined treatment often outperformed inactive care, it frequently failed to outperform aromatherapy or music alone. Adverse events, acceptability, and economic outcomes were poorly reported.

**Conclusion:**

Combined aromatherapy and music or sound showed the most consistent reductions in anxiety or distress in pediatric dentistry, MRI examinations, nursing examinations or skills training, and some perioperative and cancer-support settings. Clear benefit was not demonstrated in lithotripsy or endodontic care; in general surgery, pregnancy non-stress testing, coronary angiography, palliative care, and outpatient oncology, the combination generally did not outperform a single modality. Future adequately powered factorial trials should standardize the interventions and use prespecified between-group comparisons, longer follow-up, and systematic reporting of adverse events and costs.

**Systematic review registration:**

https://www.crd.york.ac.uk/PROSPERO/view/CRD42024570691, CRD42024570691.

## 1 Introduction

Emotional distress is not confined to psychiatric practice. It appears before surgery, during imaging and invasive procedures, in dental clinics, throughout cancer care, and when students or clinicians are assessed under pressure. In these settings, anxiety, fear, low mood, and stress can amplify pain, increase autonomic arousal, and make a necessary procedure harder to complete (World Health Organization, 2022; World Health Organization, 2025; World Health Organization, 2022).

Medication and formal psychological treatment remain central when symptoms are severe or persistent. They are less easily deployed for brief, situation-bound distress. Sedatives and anxiolytics carry adverse-effect and monitoring burdens, while psychological care requires time, trained staff, and access that is uneven across services (Garakani et al., 2020; Melaragno, 2021; Coombs et al., 2021; Lattie et al., 2022). This has kept interest in short, nonpharmacological interventions alive.

Aromatherapy and music work through different sensory routes. Volatile plant compounds provide an olfactory stimulus linked to emotion, memory, and autonomic regulation (Farrar and Farrar, 2020; Malloggi et al., 2022; Hedigan et al., 2023; Tan et al., 2023; López et al., 2017; dos Santos et al., 2022). Music can redirect attention and alter appraisal or arousal through tempo, familiarity, and preference (de Witte et al., 2020; Lu et al., 2021; Rebecchini, 2021). Each approach has shown modest benefit in some clinical populations, but protocols vary widely. Lavender may be delivered on cotton, through a diffuser, or by another inhalation method; music may be selected by investigators or by patients, heard once or repeatedly, and played before or during a procedure.

Using both interventions together is plausible, but the design of the comparison matters. Improvement from baseline in a combined group does not show that two modalities are better than one. A comparison with usual care asks whether the package does anything; a trial that also includes aroma-only and music-only arms asks whether adding the second stimulus is justified. Expectancy, attention from staff, and spontaneous recovery may otherwise be mistaken for synergy.

The relevant studies are scattered across perioperative care, dentistry, nursing education, diagnostic services, and oncology. We therefore reviewed controlled experiments published from January 2019 through September 7, 2026. The review was designed to identify where results have been reproducible, distinguish overall from incremental benefit, and clarify the practical limits of using a combined sensory intervention in routine care.

## 2 Methods

### 2.1 Design and registration

This systematic review followed the PRISMA 2020 statement (Page et al., 2020) and the Cochrane Handbook for Systematic Reviews of Interventions (Higgins et al., 2019). The protocol was registered in PROSPERO (CRD42024570691). Meta-analysis was not planned once marked variation in populations, interventions, comparators, and outcome measures became evident.

### 2.2 Information sources and search strategy

Nine databases were searched: PubMed, Embase, CINAHL, APA PsycINFO, Scopus, Web of Science Core Collection, the Cochrane Central Register of Controlled Trials (CENTRAL), China National Knowledge Infrastructure (CNKI), and Dentistry and Oral Sciences Source. Searches covered January 1, 2019, through September 7, 2026, and were last run on September 7, 2026.

The strategy combined terms for aromatherapy, essential oils, and olfactory stimulation with terms for music, music therapy, or sound. Anxiety, stress, depression, fear, pain, and emotional distress were used as outcome concepts. Dentistry, dental anxiety, pediatric dentistry, nursing education, nursing students, and clinical nurses were added to improve retrieval in fields that are inconsistently represented in general medical databases. Controlled vocabulary and syntax were adapted for each platform. The full strategies are provided in Supplementary Table S1.

### 2.3 Eligibility criteria

Eligible participants came from clinical, educational, occupational, or community settings. A formal psychiatric diagnosis was not required, but the study had to address a defined distress context, such as anxiety, stress, fear, depressive symptoms, or emotionally salient pain. The intervention had to combine aromatherapy with music or sound, either concurrently or in sequence, in a study arm that could be evaluated separately. Comparators could include usual care, no intervention, placebo or sham treatment, standard treatment, aromatherapy alone, music or sound alone, or another active intervention.

Studies had to report at least one psychological or symptom outcome measured with a validated or clearly defined instrument, or an objective physiological or biological outcome relevant to distress. Eligible designs were parallel-group randomized trials and controlled nonrandomized or quasi-experimental studies with a concurrent comparator. Study design was classified from the reported allocation method rather than the authors’ design label; reports without a verifiable random-allocation procedure were classified as controlled nonrandomized or quasi-experimental studies. Full text had to be available in English or Chinese, with first public availability between January 1, 2019, and September 7, 2026.

We excluded studies without a combined olfactory-auditory arm, uncontrolled designs, observational and qualitative reports, case reports or series, reviews, protocols, conference abstracts, editorials, dissertations available only as abstracts, and animal studies. Multicomponent programs were excluded when acupressure, virtual reality, or another treatment prevented the effect of the aroma-music combination from being isolated. Duplicate reports were resolved by retaining the most complete account of the relevant outcomes.

### 2.4 Study selection and data extraction

Records were imported into EndNote 21 and deduplicated. X.C. and D.L. independently screened titles and abstracts, then assessed potentially eligible full texts. Disagreements were resolved by discussion, with Z.L. acting as adjudicator when required. Publication eligibility was based on the date on which a complete report first became publicly available. An article posted online before the search closed remained eligible even if database indexing occurred later.

The same reviewers independently extracted study location and setting, design, sample size, participant characteristics, aroma composition and delivery, music or sound selection, timing and dose, comparator, outcome instruments, numerical and narrative findings, adverse events, acceptability, adherence, staff and equipment requirements, and any reported resource use. Missing information was recorded as not reported rather than inferred.

### 2.5 Risk-of-bias assessment

Randomized studies were assessed with the Cochrane risk-of-bias tool for randomized trials (RoB 1) across seven domains: random sequence generation, allocation concealment, blinding of participants and personnel, blinding of outcome assessment, incomplete outcome data, selective reporting, and other bias (Higgins et al., 2019; Higgins et al., 2011). Although RoB 2 is the current Cochrane tool for outcome-specific assessment, RoB 1 was retained because this review used study-level methodological judgments within a narrative synthesis of heterogeneous psychological, symptomatic, physiological, and biological outcomes; no single outcome-specific effect estimate was selected for appraisal. The resulting ratings should therefore be interpreted as study-level judgments rather than outcome-specific RoB 2 assessments. Controlled nonrandomized or quasi-experimental studies were assessed with the JBI checklist for quasi-experimental studies (Barker et al., 2024; Aromataris et al., 2024). Two reviewers completed each assessment independently and reconciled their judgments. Risk-of-bias assessments for all 24 included studies are presented in Figure 1: RoB 1 for the 21 randomized studies and the JBI quasi-experimental checklist for the three controlled nonrandomized or quasi-experimental studies. Detailed judgments are provided in Supplementary Tables S2, S3, with domain-level summaries in Supplementary Figures S1, S2.

**Figure 1:**
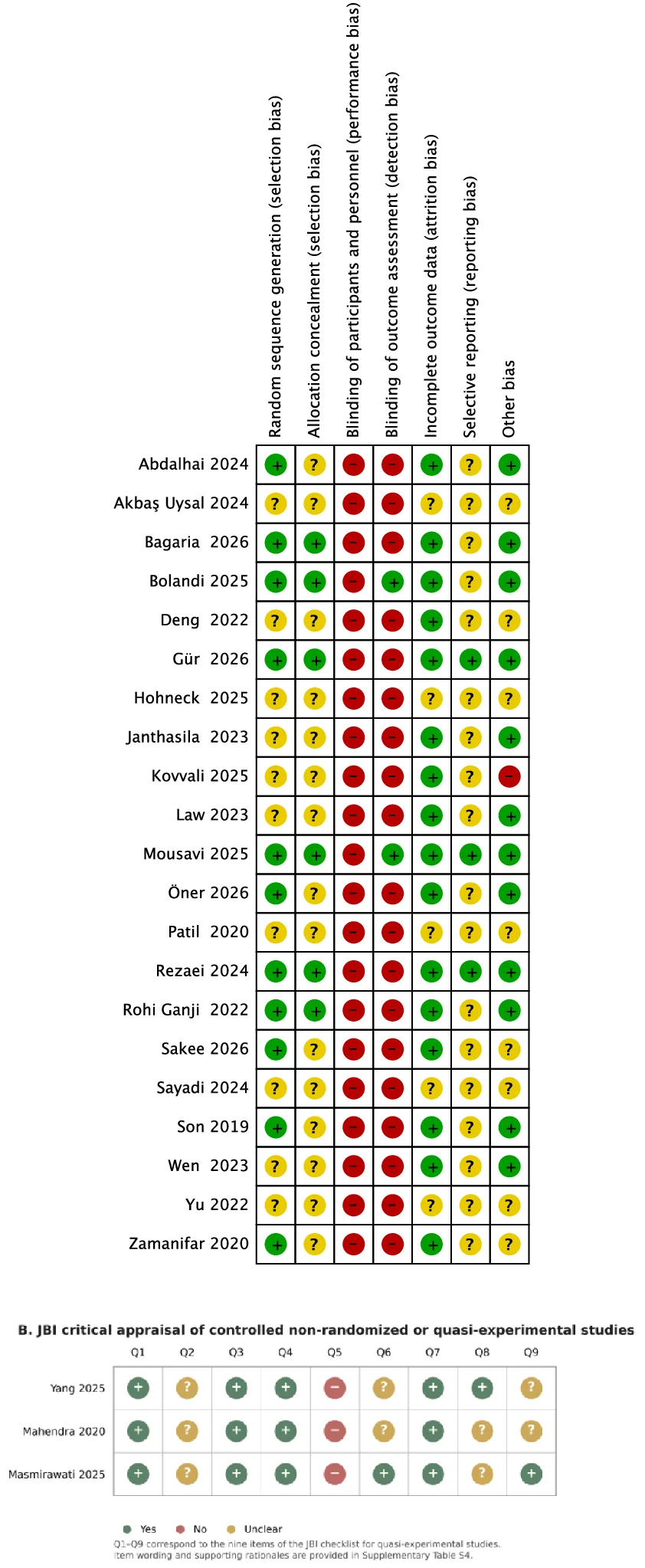
Risk-of-bias assessment of all 24 included studies. **(A)** Cochrane RoB 1 assessment of the 21 randomized studies. **(B)** JBI appraisal of the three controlled nonrandomized or quasi-experimental studies: Yang et al. (2025), Mahendra et al. (2020), and Masmirawati et al. (2025).

### 2.6 Synthesis

We used a structured narrative synthesis informed by SWiM guidance (Campbell et al., 2020). Studies were grouped into procedural and perioperative care, dentistry, nursing education or occupational stress, and cancer or palliative care. Anxiety, stress, depression, pain, fear or comfort, and physiological or biological outcomes were considered separately.

Interpretation depended on the comparator. We recorded whether the combined arm improved over time, whether it outperformed inactive or usual care, and whether it outperformed aromatherapy or music alone. Synergy was not inferred from within-group change or from superiority to an inactive control. Evidence of incremental benefit required a statistically significant direct comparison with the relevant single-modality arm.

## 3 Results

### 3.1 Study selection

Database searches identified 14,052 records, and supplementary web searches of journal and publisher websites and other academic platforms identified 40 additional records. After removal of 11,610 duplicate records, 2,482 records were screened and 2,404 were excluded by title or abstract. Of 78 full-text reports assessed, 54 were excluded because they lacked an eligible distress outcome (*n* = 21), lacked an eligible change in a distress-related outcome (*n* = 14), or did not contain an isolatable combined aromatherapy-music or sound arm (*n* = 19), leaving 24 studies in the review. The 24 included studies involved 2,530 participants (Deng et al., 2022; Sayadi Mank-Halati et al., 2024; Law et al., 2023; Rezaei et al., 2024; Rohi Ganji et al., 2022; Zamanifar et al., 2020; Wen et al., 2023; Janthasila and Keeratisiroj, 2023; Yu et al., 2022; Yang et al., 2025; Hohneck et al., 2025; Abdalhai et al., 2024; Akbaş Uysal et al., 2024; Mousavi et al., 2025; Bolandi et al., 2025; Gür and Bahcecioglu Turan, 2026; Öner et al., 2026; Kovvali et al., 2025; Mahendra et al., 2020; Bagaria et al., 2026; Sakee and Awad, 2026; Patil et al., 2020; Masmirawati et al., 2025; Son et al., 2019). One candidate report by Su and Liu combined aromatic-oil acupressure, music, and standard analgesia. Because the contribution of acupressure could not be separated from olfactory and auditory stimulation, it was excluded from the core synthesis (Su and Liu, 2023). Figure 2 summarizes study selection.

**Figure 2:**
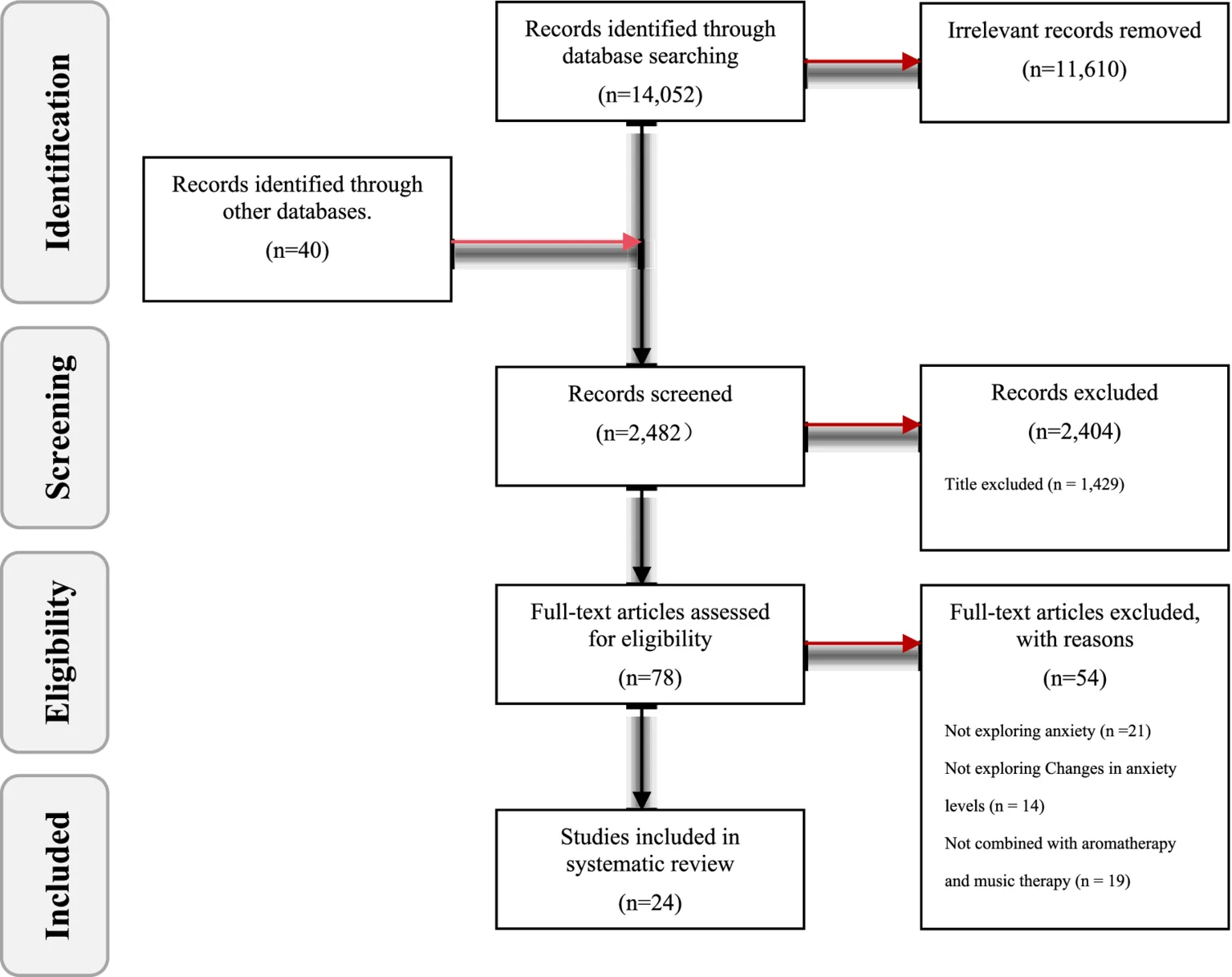
PRISMA 2020 flow diagram of study selection. Note. The flow diagram was adapted from the PRISMA 2020 statement (Page et al., 2020). The 40 additional records were identified through supplementary web searches of journal and publisher websites and other academic platforms.

### 3.2 Where and in whom the interventions were studied

The 24 reports were published between 2019 and 2026; sample sizes ranged from 36 to 236. Six studies came from Iran. Mainland China, Türkiye, and India contributed three each, and Indonesia contributed two. Thailand, Singapore, Taiwan, Germany, Syria, Egypt, and South Korea contributed one study each. No eligible study was conducted in the Americas or Oceania, and African evidence was limited to one Egyptian trial.

Nine studies examined surgery, diagnostic or cardiovascular procedures, or pregnancy-related care. Seven were conducted in dentistry, five involved students or nurses, and three involved cancer or palliative care. Twenty-one studies reported randomized allocation. Yang et al. (2025), Mahendra et al. (2020), and Masmirawati et al. (2025) were classified as controlled nonrandomized or quasi-experimental studies. Most studies were small and single-center. Table 1 gives the study-level details.

**Table 1:** Characteristics of the included studies.

**Included study**	**Country/region**	**Study setting**	**Sample size, *n***	**Participant characteristics**	**Type of emotional distress**	**Intervention and duration**	**Main outcome measures**	**Principal findings**
Deng et al. (2022)	China	Perioperative breast cancer	160	Women; mean 52.4 y	Anxiety/pain	Lavender + music; 30 min	VAS; IL-6; HMGB-1	Lower anxiety/pain and inflammatory-marker increases.
Sayadi Mank-Halati et al. (2024)	Iran	General surgery	236	Adults; mean 41.9 y	Preoperative anxiety	Rosemary + music; 30 min	STAI-S	All active arms improved; no incremental combined benefit.
Law et al. (2023)	Singapore	Lithotripsy	90	Adults; ~50 y	Pain/anxiety	Lavender + music during ESWL	STAI-S; VAS	No significant between-group benefit.
Rezaei et al. (2024)	Iran	Pregnancy NST	195	Pregnant women; mean 26.5 y	Anxiety	Lavender + nature sounds; 20 min	STAI-S; fetal NST	Both active arms improved; combination not superior.
Rohi Ganji et al. (2022)	Iran	Lithotripsy	176	Adults; ~41 y	Anxiety	Rosemary + soft music	STAI-S	No significant anxiety reduction.
Zamanifar et al. (2020)	Iran	Clinical nurses	120	Nurses; ~32 y	Occupational anxiety	Chamomile-lavender + music	BAI	Active arms reduced anxiety.
Wen et al. (2023)	China	MRI	200	Adults; mean 48.3 y	Anxiety/discomfort	Lavender + classical music; 20 min	STAI; SAS; GCQ	Combined-group anxiety reductions and comfort scores were statistically greater than in the single-modality and routine-care groups.
Janthasila and Keeratisiroj (2023)	Thailand	Pediatric dentistry	128	Children; ~11 y	Dental anxiety/fear	Diffused aroma + music; 15 min	VAS-A; CFSS-DS; FIS; vitals	Combined arm improved psychological and physiological outcomes.
Yu et al. (2022)	China	Advanced cancer	90	Adults; mean 64.4 y	Pain/anxiety/depression	Essential oils + five-element music; 28 days	SAS; SDS; VAS	Lower anxiety, depression, and pain scores were reported at later follow-up; complete pairwise effect estimates were not available for verification.
Yang et al. (2025)	Taiwan, China	Female nurses	60	Female nurses	Stress/anxiety/depression	Lavender + music; 6 weeks	NSS; BAI; BDI; vitals	No significant group-by-time superiority.
Hohneck et al. (2025)	Germany	Cancer outpatients	80	Adults	Acute distress	Scented ointment + classical music; 20 min	VAS; STAI; vascular measures	No added overall benefit over sound alone.
Abdalhai et al. (2024)	Syria	Dental local anesthesia	56	Children 6–10 y	Dental anxiety/pain	Lavender-neroli + preferred music	FIS; FLACC; vitals	Lower anxiety/vitals; pain unchanged.
Akbaş Uysal et al. (2024)	Türkiye	Palliative care	88	Adults with advanced disease	Pain/anxiety/stress	Aroma + music; 20 min daily x3	VAS; FAS; Distress Thermometer	All active arms improved; incremental benefit unclear.
Mousavi et al. (2025)	Iran	Endodontics	72	Adults	Physiological arousal	Lavender + 432-Hz music	BP; HR	No significant differences from control.
Bolandi et al. (2025)	Iran	Postoperative ICU	92	Surgical adults	Physiological instability	Rosemary + instrumental music; 30 min	RR; SpO2; vitals	Lower RR; SpO2 improved only with combination.
Gür and Bahcecioglu Turan (2026)	Türkiye	Coronary angiography	128	Adults	Pain/anxiety/fear	Aroma + music before/during CAG	VAS-P/A/F; SAI	All active arms better than control; no differences among active arms.
Öner et al. (2026)	Türkiye	Nursing injection practice	102	First-year nursing students	Anxiety	Aroma + music	Anxiety; self-esteem; BP	Anxiety/SBP improved; no combined superiority; self-esteem unchanged.
Kovvali et al. (2025)	India	Medical examinations	54	Students 18–23 y	Exam anxiety/sleep	Chamomile-lavender + classical music; 5 days	Anxiety; cognition; sleep; affect	Combined arm superior to music alone.
Mahendra et al. (2020)	Indonesia	Topical fluoride	104	Children	Dental anxiety	Sandalwood + Bossa nova	BP; pulse; anxiety	The combination group had the largest observed pre-post change; formal support for all pairwise comparisons was unclear.
Bagaria et al. (2026)	India	Restorative dentistry	45	Children 4–9 y	Anxiety/pain	Aroma + audio distraction	VAS; FLACC	Lower anxiety and pain than control.
Sakee and Awad (2026)	Egypt	Restorative dentistry	60	Children 4–7 y	Anxiety/behavior	Lavender or rosemary + music	FIS; FBRS; HR; SpO2	Both aroma-music groups improved outcomes versus tell-play-do; comparative superiority between the two aroma combinations was not established.
Patil et al. (2020)	India	Dental local anesthesia	60	Children	Anxiety/behavior	White noise + aromatherapy	Anxiety; behavior; vitals	Favorable combined-arm responses; reporting limited.
Masmirawati et al. (2025)	Indonesia	Pre-cataract surgery	36	Adults	Preoperative anxiety	Chamomile + nature sounds	Anxiety scale	Greater reduction than routine care.
Son et al. (2019)	South Korea	Nursing skills exam	98	Female nursing students	Test anxiety/stress	Marjoram-orange + classical music; 20 min	Anxiety; stress; skill score	Between-group analyses favored the combined intervention over either modality alone.

BAI, Beck Anxiety Inventory; BDI, Beck Depression Inventory; BP, blood pressure; CAG, coronary angiography; CFSS-DS, Children’s Fear Survey Schedule-Dental Subscale; ESWL, extracorporeal shockwave lithotripsy; FAS, Facial Anxiety Scale; FBRS, Frankl Behavior Rating Scale; FIS, Facial Image Scale; FLACC, Face, Legs, Activity, Cry, Consolability; GCQ, General Comfort Questionnaire; HR, heart rate; MRI, magnetic resonance imaging; NR, not reported; NST, non-stress test; SAS, Self-Rating Anxiety Scale; SDS, Self-Rating Depression Scale; SpO₂, peripheral oxygen saturation; STAI, State–Trait Anxiety Inventory; VAS, visual analogue scale.

### 3.3 Intervention and comparator characteristics

Lavender was the dominant aroma. It was used alone or blended with neroli, chamomile, or other oils. Other trials used rosemary, sandalwood, or multicomponent preparations. The auditory material ranged from participant-selected music to classical pieces, instrumental tracks, bossa nova, five-element music, natural sounds, white noise, and structured sound programs. A session usually lasted 15–30 min and occurred immediately before or during a stressful procedure. Repeated courses, extending over several days or weeks, were more common in educational and cancer-support settings.

Comparator strength varied sharply. Studies using usual care, placebo odor, or low-intensity distraction could establish whether the package was preferable to minimal care. They could not show that both sensory components were necessary. Three- or four-arm trials that included aroma-only and music-only groups provided the more informative test of incremental benefit. Intervention details are reported in Table 2.

**Table 2:** Characteristics of the multimodal sensory interventions.

**Study**	**Aromatherapy component**	**Aromatherapy delivery**	**Music or sound component**	**Multimodal delivery**	**Session duration**	**Frequency and total intervention period**	**Comparator(s)**
Deng et al. (2022)	Lavender	Inhalation	Music; genre NR	Concurrent	30 min	Single perioperative session	Aroma; music; routine care
Sayadi Mank-Halati et al. (2024)	25% rosemary; 3 drops	Cotton ball near collar	Iranian instrumental	Concurrent	30 min	Single preoperative session	Aroma; music; placebo
Law et al. (2023)	Lavender; dose NR	Inhalation	Music; details NR	During ESWL	Procedure	Single session	Analgesia; aroma + analgesia
Rezaei et al. (2024)	10% lavender; 4 drops	Scented napkin	Nature sounds	Concurrent	20 min	Single session	Aroma; water control
Rohi Ganji et al. (2022)	Rosemary; 3 drops	Inhalation	Soft music	During SWL	Procedure	Single session	Aroma; music; water control
Zamanifar et al. (2020)	Chamomile-lavender	Inhalation	Relaxing music	Concurrent	20 min	Three work shifts	Aroma; music; routine care
Wen et al. (2023)	Lavender; dose NR	Room inhalation	Pachelbel Canon	Concurrent	20 min	Single pre-MRI session	Aroma; music; routine care
Janthasila and Keeratisiroj (2023)	Oil/dose NR	Room diffuser	Music; details NR	During dental care	15 min	Single session	Aroma; music; routine care
Yu et al. (2022)	Blended oils	Inhalation	Five-element music	Concurrent	30 min	Repeated for 28 days	Aroma; music
Yang et al. (2025)	Lavender; dose NR	Inhalation	Music; details NR	Concurrent	20 min	Weekly for 6 weeks	Aroma alone
Hohneck et al. (2025)	Aurum/Lavandula ointment	Upper chest topical	Classical compilation	Concurrent	20 min	Single session	Sound alone
Abdalhai et al. (2024)	Lavender-neroli; 3 drops	Modified nasal mask	Preferred music	Before/during injection	~5 min + procedure	Single session	Empty-mask placebo
Akbaş Uysal et al. (2024)	Oil/dose NR	Inhalation	Music; details NR	Concurrent	20 min	Daily for 3 days	Aroma; music; control
Mousavi et al. (2025)	Lavender; 5 drops	Humidifier	432-Hz music	During endodontics	Procedure	Single session	Aroma; music; control
Bolandi et al. (2025)	25% rosemary	Inhalation	Instrumental music	Concurrent	30 min	Postoperative ICU session	Aroma; music; placebo
Gür and Bahcecioglu Turan (2026)	Oil/dose NR	Inhalation	Music; details NR	Before/during CAG	Procedure	Single session	Aroma; music; control
Öner et al. (2026)	Oil/dose NR	Inhalation	Music-assisted aroma	Before skills practice	NR	Single session	Aroma; control
Kovvali et al. (2025)	Chamomile-lavender	Inhalation	Moonlight Sonata	Concurrent	20 min	Daily for 5 days	Music; control
Mahendra et al. (2020)	Sandalwood	Inhalation	Bossa nova	During fluoride care	NR	Single session	Aroma; music; control
Bagaria et al. (2026)	Oil/dose NR	Inhalation	Audio distraction	During restoration	NR	Single session	Audiovisual + aroma; control
Sakee and Awad (2026)	Lavender or rosemary	Inhalation	Music	During restoration	NR	Single session	Tell-play-do
Patil et al. (2020)	Oil/dose NR	Inhalation	White noise	During local anesthesia	NR	Single session	VR; audio; routine care
Masmirawati et al. (2025)	Chamomile	Inhalation	Nature sounds	Pre-cataract	NR	Single session	Routine care
Son et al. (2019)	Marjoram-orange; 3 drops	Aroma lamp	Moonlight Sonata	Concurrent	20 min	Single pre-exam session	Aroma; music

CAG, coronary angiography; ESWL, extracorporeal shockwave lithotripsy; ICU, intensive care unit; MRI, magnetic resonance imaging; NR, not reported; NST, non-stress test.

### 3.4 Findings by clinical setting

#### 3.4.1 Procedural, perioperative, and cardiovascular care

Deng et al. reported significantly greater improvements in anxiety and pain with the combined intervention than with either single modality or routine care; perioperative increases in interleukin-6 and high-mobility group box 1 were also smaller in the combined group (Deng et al., 2022). Wen et al. (2023) reported significantly greater reductions in anxiety and higher comfort scores in the combined group than in the aromatherapy, music, and routine-care groups. Masmirawati et al. (2025) reported lower preoperative anxiety with chamomile aromatherapy plus natural sounds than with routine care before cataract surgery.

Other procedural studies did not establish consistent superiority of the combined intervention. In general surgery, rosemary aromatherapy, music, and their combination each reduced preoperative anxiety compared with placebo, but the report did not establish an incremental advantage of the combined intervention (Sayadi Mank-Halati et al., 2024). During pregnancy non-stress testing, lavender alone and lavender plus natural sounds improved state anxiety and fetal test parameters relative to control, without evidence that the combination was superior to aromatherapy alone (Rezaei et al., 2024). In coronary angiography, aromatherapy, music, and the combined intervention reduced pain, anxiety, and fear compared with control, but the active groups did not differ significantly (Gür and Bahcecioglu Turan, 2026). In postoperative intensive care, aromatherapy reduced heart rate and respiratory rate, whereas the combined intervention reduced respiratory rate and increased oxygen saturation; blood pressure did not differ significantly between groups (Bolandi et al., 2025).

The two lithotripsy trials did not demonstrate a clear benefit. Law et al. (2023) found that adding aromatherapy and music to standard analgesia did not significantly reduce pain or anxiety, while Rohi Ganji et al. (2022) found no significant reduction in anxiety with aromatherapy, music, or their combination.

#### 3.4.2 Dentistry

Six of the seven dental studies involved children. Janthasila and Keeratisiroj (2023) reported a significant interaction between music and aromatherapy for dental anxiety and fear, together with favorable physiological changes. Mahendra et al. (2020) reported that the combination group had the largest observed pre-post changes in blood pressure and pulse, but the report did not clearly establish all pairwise between-group comparisons. In Abdalhai et al. (2024), lavender-neroli oil combined with preferred music reduced anxiety, heart rate, and blood pressure during local-anesthetic administration, whereas pain and oxygen saturation did not differ significantly. Bagaria et al. (2026) reported lower anxiety and procedural pain with a package that also included visual distraction. Sakee and Awad (2026) reported improvements in anxiety, behavior, heart rate, and oxygen saturation with both aroma-music interventions relative to tell-play-do, while Patil et al. (2020) reported improvements in anxiety and disruptive behavior with white noise plus aromatherapy but provided limited physiological detail. Because several packages contained additional distraction components and some reports provided sparse methods, the independent contribution of aromatherapy plus music could not always be isolated.

The only adult endodontic trial recorded numerical decreases in blood pressure and heart rate, but neither the within-group changes nor the between-group differences were statistically significant (Mousavi et al., 2025). Thus, the positive dental findings were concentrated in pediatric studies; evidence for adults was limited to one physiologically negative trial.

#### 3.4.3 Nursing education, students, and occupational stress

Two studies reported incremental benefit under short-term academic or performance pressure. Son et al. found that combined aromatherapy and music produced greater improvements in test anxiety, state anxiety, stress, and intravenous catheterization performance than either intervention alone (Son et al., 2019). Kovvali et al. (2025) reported that adding aromatherapy to music improved examination anxiety, cognitive performance, sleep quality, and positive affect compared with music alone; a significant benefit for negative affect was not reported.

Findings were less consistent in occupational or routine training contexts. Zamanifar et al. (2020) observed anxiety reductions with music, chamomile-lavender aromatherapy, and their combination compared with control, but the combined intervention was not superior to either single modality. Among nursing students practicing injections, aromatherapy alone and aromatherapy plus music reduced anxiety and systolic blood pressure, whereas self-esteem did not change significantly and the combined intervention showed no clear incremental advantage (Öner et al., 2026). Yang et al. (2025) reported reductions over time in stress, anxiety, and depression among female nurses, but did not demonstrate a consistent group-by-time advantage for the combined intervention; systolic blood pressure, diastolic blood pressure, and pulse were also assessed.

#### 3.4.4 Cancer and palliative care

Among patients with advanced cancer, the report described lower anxiety, depression, and pain scores after repeated aromatherapy combined with five-element music than after either component alone; however, the available reporting did not permit independent verification of all pairwise effect estimates (Yu et al., 2022). In palliative care, pain, anxiety, and stress decreased over 3 days in the aromatherapy, music, and combined groups, but the combined arm did not show a consistent advantage over the single-modality arms (Akbaş Uysal et al., 2024). Hohneck et al. (2025) observed similar improvement in psychophysiological measures with sound alone and sound plus aromatherapy, without a substantial added benefit from aromatherapy; some cardiovascular findings favored sound alone.

### 3.5 Distribution of findings across populations and settings

Across settings, favorable results were more frequent when the combined intervention was compared with inactive or routine care than when it was compared with aromatherapy or music alone. Evidence of incremental benefit was reported in MRI, several pediatric dental studies, nursing examinations or skills testing, perioperative breast cancer care, and one repeated advanced-cancer program (Deng et al., 2022; Wen et al., 2023; Janthasila and Keeratisiroj, 2023; Yu et al., 2022; Kovvali et al., 2025; Mahendra et al., 2020; Son et al., 2019). By contrast, general surgery, pregnancy non-stress testing, coronary angiography, clinical nursing, female nurses during the COVID-19 pandemic, palliative care, and outpatient oncology generally showed similar improvement across active interventions or no reliable added effect from the second modality (Sayadi Mank-Halati et al., 2024; Rezaei et al., 2024; Zamanifar et al., 2020; Yang et al., 2025; Hohneck et al., 2025; Akbaş Uysal et al., 2024; Gür and Bahcecioglu Turan, 2026; Öner et al., 2026). Study-level patient-reported and physiological or biological outcomes are summarized in Table 3 and Figure 3 displays the direction of reported effects and distinguishes comparisons with inactive care from comparisons with a single sensory intervention.

**Table 3:** Summary of psychological, symptomatic, physiological, and biological outcomes.

**Study**	**Patient-reported outcomes**	**Physiological/biological outcomes**	**Main patient-reported findings**	**Main physiological/biological findings**
Deng et al. (2022)	Anxiety; pain	IL-6; HMGB-1	The trial reported significantly greater improvements in anxiety and pain with the combined intervention than with either single modality or routine care.	The trial reported smaller perioperative increases in IL-6 and HMGB-1 in the combined group than in the comparison groups.
Sayadi Mank-Halati et al. (2024)	Anxiety	Not assessed	Aromatherapy, music, and the combined intervention reduced anxiety versus placebo; an incremental advantage of the combination was not established.	Not assessed or not reported.
Law et al. (2023)	Anxiety; pain	Not assessed	Adding aromatherapy and music to standard analgesia did not significantly reduce pain or anxiety.	Not assessed or not reported.
Rezaei et al. (2024)	State anxiety	Fetal NST parameters	Aromatherapy alone and aromatherapy with music reduced state anxiety versus control; the combined intervention was not superior to aromatherapy alone.	Both active interventions improved NST results, without an incremental benefit from music.
Rohi Ganji et al. (2022)	Anxiety	Not assessed	Aromatherapy, music, and their combination did not reduce anxiety during lithotripsy.	Not assessed or not reported.
Zamanifar et al. (2020)	Anxiety	Not assessed	Music, chamomile–lavender aromatherapy, and their combination reduced anxiety versus control; the combined arm was not superior to either single modality.	Not assessed or not reported.
Wen et al. (2023)	Anxiety; comfort	Not assessed	The combined group had significantly greater anxiety reductions and higher comfort scores than the aromatherapy, music, and routine-care groups.	Not assessed or not reported.
Janthasila and Keeratisiroj (2023)	Dental anxiety; dental fear	HR; BP; SpO₂	Factorial analysis showed a significant music-by-aromatherapy interaction for dental anxiety and fear.	The combined group showed favorable HR, BP, and SpO2 changes; the interaction was statistically significant for SpO2.
Yu et al. (2022)	Anxiety; depression; pain	Not assessed	Lower anxiety, depression, and pain scores were reported at later follow-up; complete pairwise effect estimates were not available for verification.	Not assessed or not reported.
Yang et al. (2025)	Stress; anxiety; depression	SBP; DBP; pulse rate	Both groups improved over time; the available analyses did not establish consistent superiority of aromatherapy plus music over aromatherapy alone.	Physiological measures generally improved within groups, but a consistent incremental effect of music was not established.
Hohneck et al. (2025)	Anxiety; stress; pain	Cardiovascular parameters	Sound alone and sound plus aromatherapy improved psychophysiological outcomes; aromatherapy provided no substantial added benefit.	Cardiovascular improvement was numerically greater with sound alone, without a significant between-group difference.
Abdalhai et al. (2024)	Dental anxiety; pain	HR; SBP; DBP; SpO₂	The combined intervention reduced dental anxiety versus placebo; pain did not differ between groups.	HR and BP were lower in the intervention group; SpO₂ did not differ.
Akbaş Uysal et al. (2024)	Pain; anxiety; stress	Not assessed	Music, aromatherapy, and their combination reduced pain, anxiety, and stress; a stable incremental benefit of the combination was not demonstrated.	Not assessed or not reported.
Mousavi et al. (2025)	Not assessed	SBP; DBP; HR	Not assessed or not reported.	BP and HR decreased numerically in the intervention groups, but differences from control were not significant.
Bolandi et al. (2025)	Not assessed	HR; respiratory rate; BP; SpO₂	Not assessed or not reported.	Aromatherapy reduced HR and respiratory rate; the combined intervention reduced respiratory rate and increased SpO₂; BP did not change significantly.
Gür and Bahcecioglu Turan (2026)	Pain; anxiety; fear	Not assessed	All active interventions reduced pain, anxiety, and fear versus control; differences among the active arms were not significant.	Not assessed or not reported.
Öner et al. (2026)	Anxiety; self-esteem	SBP; DBP; pulse rate	Both intervention groups reduced anxiety versus control; self-esteem was unchanged and the combined intervention was not superior.	Both intervention groups reduced SBP; music provided no added physiological benefit.
Kovvali et al. (2025)	Exam anxiety; cognition; sleep quality; positive and negative affect	Not assessed	The combined intervention improved exam anxiety, cognition, sleep quality, and positive affect versus music alone; no significant benefit for negative affect was reported.	Not assessed or not reported.
Mahendra et al. (2020)	Dental anxiety	BP; pulse rate	The combination group showed the largest observed pre-post reduction; the report did not clearly establish all pairwise between-group comparisons.	BP and pulse improved in the active groups; the combination group had the largest observed change, with pairwise significance unclear.
Bagaria et al. (2026)	Dental anxiety; procedural pain	Not assessed	Aromatherapy with audio or audiovisual distraction reduced anxiety and procedural pain versus control.	Not assessed or not reported.
Sakee and Awad (2026)	Dental anxiety; dental behavior	HR; SpO₂	Both aroma-music interventions improved anxiety and behavior versus tell-play-do; superiority of one aroma-music combination over the other was not established.	HR and SpO₂ favored both aroma–music groups across treatment stages.
Patil et al. (2020)	Dental anxiety; disruptive behavior	Vital signs	White noise plus aromatherapy produced favorable anxiety and behavior responses, although comparative reporting was limited.	Favorable physiological responses were reported, but detail was limited.
Masmirawati et al. (2025)	Preoperative anxiety	Not assessed	Chamomile aromatherapy with natural sounds reduced anxiety more than routine care.	Not assessed or not reported.
Son et al. (2019)	Test anxiety; state anxiety; stress; nursing-skill performance	Not assessed	Between-group analyses favored the combined intervention over aromatherapy and music alone for anxiety, stress, and nursing-skill performance.	Not assessed or not reported.

BP, blood pressure; DBP, diastolic blood pressure; HMGB-1, high-mobility group box 1; HR, heart rate; IL-6, interleukin-6; NST, non-stress test; SBP, systolic blood pressure; SpO₂, peripheral oxygen saturation.

**Figure 3:**
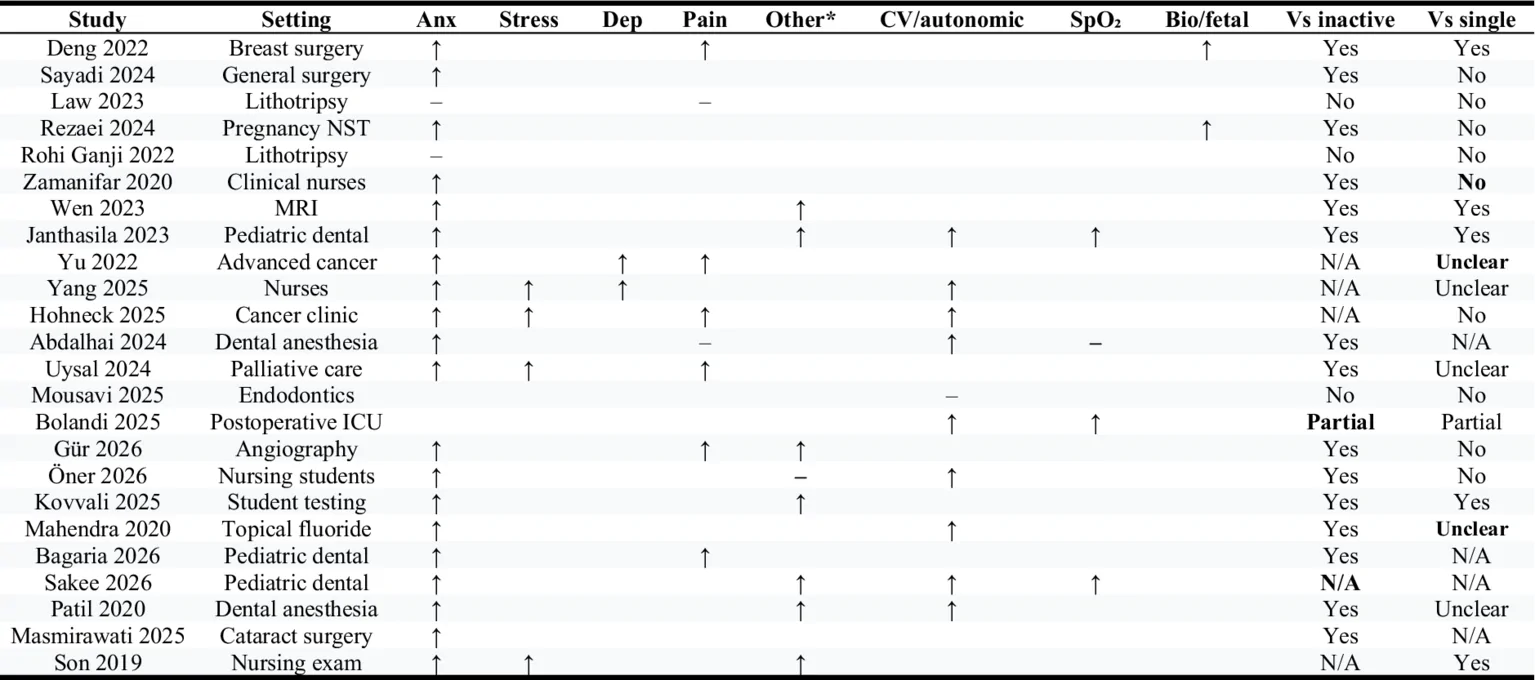
Direction of reported effects by setting and comparator. ↑, favorable statistically significant effect reported; −, no statistically significant benefit; blank, outcome not assessed or not reported. *Other outcomes: comfort (Wen), dental fear (Janthasila and Gür), self-esteem (Öner), cognition/sleep/affect (Kovvali), dental behavior (Sakee and Patil), and nursing-skill performance (Son). In the comparator columns, Yes denotes a statistically supported direct comparison or interaction favoring the combined intervention; No denotes no statistically significant added benefit; Partial denotes outcome-dependent findings; Unclear denotes a descriptive difference without clearly reported formal comparative support; and N/A denotes that the relevant comparator was absent. Arrows indicate direction only and are not effect-size estimates.

No study developed or validated a predictive responder profile. The positive evidence was generated mainly in studies enrolling children or adults with brief anticipatory or situational distress, particularly in pediatric dental care, MRI, and short educational assessments. Evidence was less consistent in procedures dominated by continuous pain or machine-related stimulation, such as lithotripsy, and when an active single-modality comparator already produced improvement. This distribution describes the populations and settings represented in the current evidence base; it does not demonstrate effect modification or identify individuals who are more likely to benefit.

### 3.6 Safety, acceptability, and implementation

No serious intervention-related adverse event was reported. That finding is difficult to interpret because most studies did not collect headache, nausea, airway irritation, fragrance intolerance, skin reactions, auditory discomfort, or reasons for withdrawal systematically. Delivery generally required cotton pads or a diffuser and a standard audio device. Sessions were brief and were usually administered by nurses, dental staff, or research personnel. None of the studies included a formal cost-effectiveness, budget-impact, or staff-time analysis.

### 3.7 Methodological quality

Common concerns were inadequate reporting of allocation concealment, the impossibility of participant blinding, reliance on self-report, small samples, and absent or inaccessible prespecified analysis plans. The three controlled nonrandomized or quasi-experimental studies also raised questions about baseline comparability, allocation procedures, and residual confounding (Yang et al., 2025; Mahendra et al., 2020; Masmirawati et al., 2025). Several newly included reports appeared in journals with limited database coverage or sparse methods sections. Their findings were retained, but they were not given the same interpretive weight as well-reported randomized trials with single-modality comparators.

## 4 Discussion

### 4.1 Principal interpretation

This review found a treatment signal, but not a general rule. Combined aroma and music reduced situational distress in several settings; the same package failed in others, and an effective single modality was often enough. The most coherent positive pattern involved pediatric dental care, MRI, short educational assessments, and a small number of repeated supportive-care programs. Lithotripsy and adult endodontic physiology sat at the opposite end of the spectrum.

The distinction between overall and incremental benefit is central. A combined arm may be better than routine care even when the second component contributes little. This was visible in coronary angiography and palliative care, where active groups improved but did not separate reliably from one another (Akbaş Uysal et al., 2024; Gür and Bahcecioglu Turan, 2026). The question for practice is therefore not simply whether patients feel better after aroma and music. It is whether the extra component improves an outcome enough to justify another preference check, another piece of equipment, and another possible source of discomfort.

### 4.2 Relation to earlier reviews and contribution of this study

Earlier reviews have examined inhaled essential oils or music as separate approaches and reached broadly favorable but heterogeneous conclusions (Hedigan et al., 2023; Tan et al., 2023; de Witte et al., 2020; Lu et al., 2021; Czakert et al., 2022; Yoo and Park, 2023; Kulakaç and Çilingir, 2024; Park et al., 2024). They do not resolve the component question. By bringing dentistry and nursing education into the same synthesis as perioperative and cancer care, the present review shows that setting changes the interpretation at least as much as the choice of oil or music.

The advance here is analytic rather than technological. We separated change within the combined arm, benefit over inactive care, and benefit over a single modality. Once those comparisons were disentangled, the apparent consistency of the literature diminished. A small set of trials supports adding the second modality; a larger set supports sensory care in general.

### 4.3 Why effects differed

Olfactory input reaches limbic and hypothalamic networks involved in emotional salience, memory, and autonomic control. Lavender constituents such as linalool have been proposed to influence inhibitory signaling, although human mediation evidence remains limited (López et al., 2017; dos Santos et al., 2022). Music can reduce threat appraisal, occupy attention, and support parasympathetic recovery, especially when tempo and familiarity suit the listener (Rebecchini, 2021). Concordant stimuli may reinforce a sense of safety. A disliked odor, unfamiliar music, excessive intensity, or headphones that obstruct communication can do the reverse.

The clinical trigger also matters. Anticipatory anxiety before imaging, injection, or an examination is partly sustained by attention and expectation, which are reasonable targets for sensory modulation. Lithotripsy delivers ongoing pain, vibration, and noise. Those inputs may keep sympathetic arousal high despite a short relaxation intervention. Repeated sessions in supportive care may acquire a conditioned calming effect; a single exposure during an intense procedure has less opportunity to do so.

Methodological choices add further variation. An inactive comparator makes a difference easier to detect. When aromatherapy or music alone is effective, the remaining margin for improvement is smaller and requires a larger trial. Baseline distress, age, cultural familiarity, analgesic use, dose, timing, and outcome assessment can all shift the observed effect. The few biomarker findings are useful leads, not proof that autonomic or hypothalamic–pituitary–adrenal pathways mediated symptom change.

### 4.4 Clinical implications

The available evidence has primarily been generated among patients or students experiencing mild-to-moderate, time-limited anticipatory or situational distress, including those undergoing pediatric dental procedures, MRI, and brief skills assessments. This pattern should not be interpreted as a validated responder profile or as evidence that particular individual characteristics predict greater benefit. In the settings represented by the current studies, the intervention may be offered as an optional, preference-sensitive adjunct when both sensory components are acceptable. Severe anxiety, acute psychiatric symptoms, and procedures dominated by substantial pain require established treatment first.

Implementation need not be elaborate. Before the procedure, staff can assess distress and ask whether the person wants scent, music, both, or neither. Screening should cover fragrance allergy, asthma or airway sensitivity, migraine triggers, skin sensitivity, and hearing limitations. A low-intensity aroma and familiar or patient-selected audio can then be offered for about 15–30 min without compromising conversation or monitoring. Ventilation, infection control, and the ability to stop immediately are basic safeguards.

A combined protocol should not become the default merely because it appears more comprehensive. If music alone is preferred or produces the same result, adding fragrance creates no demonstrated clinical gain. The reverse also applies.

### 4.5 Cost and feasibility

The equipment burden is modest: a reusable audio device, delivery materials, and small quantities of quality-controlled oil. Nurses or dental staff can usually deliver a simple protocol after brief training, which makes use in waiting areas, procedure rooms, bedside care, and simulation laboratories feasible.

Low purchase cost is not the same as cost-effectiveness. Screening and documentation take staff time; oils require procurement and storage; shared equipment must be cleaned; and ventilation or fragrance-free policies may restrict use. No included study tested whether the intervention reduces medication, sedation, procedure interruption, or staff workload. At present, “low-resource” is defensible; “cost-saving” is not.

### 4.6 Limitations and research priorities

The evidence base is heterogeneous and mostly small. Oils, concentrations, audio material, exposure schedules, comparators, and outcome tools varied too much for a credible pooled estimate. Participant blinding was rarely possible, and expectancy may have influenced self-reported outcomes. Follow-up was generally short. Evidence came largely from Asia and the Middle East, where scent and music preferences may not generalize to other populations. Adverse-event and economic reporting was particularly thin.

Future trials should use a four-arm factorial design whenever the aim is to test a combination: aromatherapy, music, both, and control. Sample-size calculations should be based on the smaller incremental effect, not the expected difference from usual care. Reports need the oil composition and concentration, delivery distance, audio source and volume, selection procedure, fidelity, co-interventions, and adherence. Prespecified stratification by age, baseline distress, pain intensity, setting, and sensory preference would be more informative than *post hoc* claims about responders.

Mechanistic work should pair validated distress measures with heart-rate variability, cortisol, or another prespecified marker and test mediation directly. Pragmatic trials should record procedure completion, analgesic or sedative use, staff time, consumables, adverse events, and patient refusal. Those outcomes will determine whether a promising brief intervention improves care rather than simply adding activity.

## 5 Conclusion

Overall, the intervention appears to be a context-dependent adjunct rather than a consistently superior multimodal treatment. Its clinical value may lie in providing a simple, nonpharmacological option for brief anticipatory distress, whereas adding a second sensory component offers limited advantage when one modality is already effective or when procedural pain predominates. Future research should determine whether the combined approach provides clinically meaningful incremental benefit and whether that benefit justifies the additional implementation burden.

## Data Availability

The original contributions presented in the study are included in the article/Supplementary material, further inquiries can be directed to the corresponding author/s.
