# Correction: Combined aromatherapy and music or sound for emotional distress: a systematic review of controlled studies published between 2019 and 2026

**DOI:** 10.3389/fpsyg.2026.2023880

**Published:** 2026-10-23

**Authors:** Haojian Zhai, Xinyue Chen, Dong Lv, Amy Jing-Wen Yin, Wenge Zhang, Yunkai Wang, Qiang Li, Zhixiong Lin

**Affiliations:** 1 Department of Psychiatry, Affiliated Hospital of Guangdong Medical University, Zhanjiang, Guangdong, China; 2 Neuromodulation Center, Dongguan Seventh People's Hospital, Dongguan, Guangdong, China; 3 School of Rehabilitation Science, Shanghai University of Traditional Chinese Medicine, Shanghai, China; 4 College of Art and Design, Lingnan Normal University, Zhanjiang, Guangdong, China

**Keywords:** anxiety, aromatherapy, emotional distress, multisensory intervention, music therapy, systematic review

Author Dong Lv was erroneously assigned as equal contributing author. The designation was also erroneously missing from Haojian Zhai. The following is correct:

Haojian Zhai^1, 2†^, Xinyue Chen^1†^, Dong Lv^1^, Amy Jing-Wen Yin^1^, Wenge Zhang^1^, Yunkai Wang^3*^, Qiang Li^4*^ and Zhixiong Lin^1*^

^†^These authors have contributed equally to this work

The original version of this article has been updated.

