# Supplementary material for: Combined aromatherapy and music or sound for emotional distress: a systematic review of controlled studies published between 2019 and 2026
Source: Front Psychol. 2026 Oct 8;17:1955987. doi: 10.3389/fpsyg.2026.1955987 (PMC13687314; doi:10.3389/fpsyg.2026.1955987)
Supplement: Supplementary file 1 [file Supplementary_file_1.DOCX]

Supplementary Material

Combined Aromatherapy and Music or Sound for Emotional Distress: A Systematic Review of Controlled Studies Published Between 2019 and 2026

Search updated through 7 September 2026

# **Contents**

**Supplementary Table S1. Database-specific search strategies**

**Supplementary Table S2. Detailed Cochrane RoB 1 assessment for randomized studies (21 randomized studies)**

**Supplementary Table S3. JBI appraisal of controlled nonrandomized/quasi-experimental studies (3 controlled nonrandomized or quasi-experimental studies)**

**Supplementary Figure S1. Distribution of RoB 1 judgements by domain**

**Supplementary Figure S2. JBI appraisal summary**

# Supplementary Table S1. Database-specific search strategies

The searches combined a core intervention-outcome strategy with targeted sensitivity searches for dentistry and nursing education. Syntax was adapted to each platform. No language filter was applied at the search stage; full-text eligibility was restricted to English or Chinese reports.

| Database and platform | Last searched | Coverage | Complete strategy |
| --- | --- | --- | --- |
| PubMed/MEDLINE | 7 September 2026 | 1 January 2019-7 September 2026 | (("Aromatherapy"[Mesh] OR aromatherap*[tiab] OR "essential oil"[tiab] OR "essential oils"[tiab] OR lavender[tiab] OR neroli[tiab] OR rosemary[tiab] OR sandalwood[tiab] OR chamomile[tiab] OR olfactory[tiab] OR fragrance*[tiab]) AND ("Music Therapy"[Mesh] OR music*[tiab] OR "sound therapy"[tiab] OR "natural sound*"[tiab] OR "white noise"[tiab] OR "audio analgesia"[tiab]) AND ("Anxiety"[Mesh] OR anxi*[tiab] OR stress*[tiab] OR distress*[tiab] OR depress*[tiab] OR fear*[tiab] OR pain[tiab] OR comfort[tiab])) OR ((aromatherap*[tiab] OR "essential oil*"[tiab]) AND (music*[tiab] OR sound*[tiab]) AND (dent*[tiab] OR "nursing education"[tiab] OR "nursing student*"[tiab] OR nurse*[tiab])) Filters: publication date 2019/01/01-2026/09/07; humans. |
| Embase (Elsevier) | 7 September 2026 | 1 January 2019-7 September 2026 | (('aromatherapy'/exp OR aromatherap*:ti,ab,kw OR 'essential oil'/exp OR 'essential oil*':ti,ab,kw OR lavender:ti,ab,kw OR neroli:ti,ab,kw OR rosemary:ti,ab,kw OR sandalwood:ti,ab,kw OR chamomile:ti,ab,kw OR olfactory:ti,ab,kw OR fragrance*:ti,ab,kw) AND ('music therapy'/exp OR music*:ti,ab,kw OR 'sound therapy':ti,ab,kw OR 'natural sound*':ti,ab,kw OR 'white noise':ti,ab,kw OR 'audio analgesia':ti,ab,kw) AND ('anxiety'/exp OR anxi*:ti,ab,kw OR stress*:ti,ab,kw OR distress*:ti,ab,kw OR depress*:ti,ab,kw OR fear*:ti,ab,kw OR pain:ti,ab,kw OR comfort:ti,ab,kw)) OR ((aromatherap*:ti,ab,kw OR 'essential oil*':ti,ab,kw) AND (music*:ti,ab,kw OR sound*:ti,ab,kw) AND (dent*:ti,ab,kw OR 'nursing education':ti,ab,kw OR 'nursing student*':ti,ab,kw OR nurse*:ti,ab,kw)) AND [2019-2026]/py |
| CINAHL Complete (EBSCOhost) | 7 September 2026 | 1 January 2019-7 September 2026 | ((MH "Aromatherapy+" OR MH "Oils, Essential+" OR TX aromatherap* OR TX "essential oil*" OR TX lavender OR TX neroli OR TX rosemary OR TX sandalwood OR TX chamomile OR TX olfactory OR TX fragrance*) AND (MH "Music Therapy+" OR TX music* OR TX "sound therapy" OR TX "natural sound*" OR TX "white noise" OR TX "audio analgesia") AND (MH "Anxiety+" OR MH "Stress, Psychological+" OR TX anxi* OR TX stress* OR TX distress* OR TX depress* OR TX fear* OR TX pain OR TX comfort)) OR ((TX aromatherap* OR TX "essential oil*") AND (TX music* OR TX sound*) AND (TX dent* OR TX "nursing education" OR TX "nursing student*" OR TX nurse*)) Limiters: published date 20190101-20260907. |
| APA PsycINFO (EBSCOhost) | 7 September 2026 | 1 January 2019-7 September 2026 | ((DE "Aromatherapy" OR TX aromatherap* OR TX "essential oil*" OR TX lavender OR TX neroli OR TX rosemary OR TX sandalwood OR TX chamomile OR TX olfactory OR TX fragrance*) AND (DE "Music Therapy" OR TX music* OR TX "sound therapy" OR TX "natural sound*" OR TX "white noise" OR TX "audio analgesia") AND (DE "Anxiety" OR DE "Psychological Stress" OR TX anxi* OR TX stress* OR TX distress* OR TX depress* OR TX fear* OR TX pain OR TX comfort)) OR ((TX aromatherap* OR TX "essential oil*") AND (TX music* OR TX sound*) AND (TX dent* OR TX "nursing education" OR TX "nursing student*" OR TX nurse*)) Limiters: publication year 2019-2026; search closed 7 September 2026. |
| Scopus | 7 September 2026 | 1 January 2019-7 September 2026 | TITLE-ABS-KEY((aromatherap* OR "essential oil*" OR lavender OR neroli OR rosemary OR sandalwood OR chamomile OR olfactory OR fragrance*) AND (music* OR "sound therapy" OR "natural sound*" OR "white noise" OR "audio analgesia") AND (anxi* OR stress* OR distress* OR depress* OR fear* OR pain OR comfort)) OR TITLE-ABS-KEY((aromatherap* OR "essential oil*") AND (music* OR sound*) AND (dent* OR "nursing education" OR "nursing student*" OR nurse*)) AND PUBYEAR > 2018 AND PUBYEAR < 2027; online-first records checked through 7 September 2026. |
| Web of Science Core Collection | 7 September 2026 | 1 January 2019-7 September 2026 | TS=((aromatherap* OR "essential oil*" OR lavender OR neroli OR rosemary OR sandalwood OR chamomile OR olfactory OR fragrance*) AND (music* OR "sound therapy" OR "natural sound*" OR "white noise" OR "audio analgesia") AND (anxi* OR stress* OR distress* OR depress* OR fear* OR pain OR comfort)) OR TS=((aromatherap* OR "essential oil*") AND (music* OR sound*) AND (dent* OR "nursing education" OR "nursing student*" OR nurse*)); PY=2019-2026; final date check 7 September 2026. |
| Cochrane CENTRAL | 7 September 2026 | 1 January 2019-7 September 2026 | ([mh Aromatherapy] OR [mh "Oils, Volatile"] OR aromatherap*:ti,ab,kw OR "essential oil*":ti,ab,kw OR lavender:ti,ab,kw OR neroli:ti,ab,kw OR rosemary:ti,ab,kw OR sandalwood:ti,ab,kw OR chamomile:ti,ab,kw OR olfactory:ti,ab,kw OR fragrance*:ti,ab,kw) AND ([mh "Music Therapy"] OR music*:ti,ab,kw OR "sound therapy":ti,ab,kw OR "natural sound*":ti,ab,kw OR "white noise":ti,ab,kw OR "audio analgesia":ti,ab,kw) AND ([mh Anxiety] OR anxi*:ti,ab,kw OR stress*:ti,ab,kw OR distress*:ti,ab,kw OR depress*:ti,ab,kw OR fear*:ti,ab,kw OR pain:ti,ab,kw OR comfort:ti,ab,kw); publication year 2019-2026. |
| CNKI | 7 September 2026 | 1 January 2019-7 September 2026 | Chinese-language subject search using the Chinese equivalents of: Subject=(aromatherapy OR aroma treatment OR aromatic nursing OR essential oil OR fragrance OR lavender OR neroli OR rosemary OR sandalwood OR chamomile) AND Subject=(music therapy OR music treatment OR music intervention OR sound therapy OR natural sounds OR white noise OR audio analgesia) AND Subject=(anxiety OR stress OR emotional distress OR depression OR fear OR pain OR comfort). Sensitivity search: aromatherapy/essential oils AND music/sound AND dentistry/pediatric dentistry/nursing education/nursing students/nurses. Publication date: 2019-01-01 to 2026-09-07. |
| Dentistry & Oral Sciences Source (EBSCOhost) | 7 September 2026 | 1 January 2019-7 September 2026 | ((TX aromatherap* OR TX "essential oil*" OR TX lavender OR TX neroli OR TX rosemary OR TX sandalwood OR TX chamomile OR TX fragrance*) AND (TX music* OR TX "sound therapy" OR TX "natural sound*" OR TX "white noise" OR TX "audio analgesia") AND (TX anxi* OR TX fear* OR TX stress* OR TX pain OR TX behavior* OR TX behaviour*)) AND (TX dent* OR TX oral OR TX odont*); published date 20190101-20260907. |

# Supplementary Table S2. Detailed Cochrane RoB 1 assessment for randomized studies (21 randomized studies)

The judgements in this table are study-level assessments anchored to the main emotional-distress and related outcomes reported in each study. Where studies included both subjective and objective outcomes, differences in susceptibility to performance or detection bias were considered in the supporting rationale, while one study-level judgement was retained for each RoB 1 domain. Low risk, High risk, and Unclear risk correspond to the three RoB 1 judgement categories. This table includes the 21 studies for which randomized allocation was reported and sufficiently identifiable.

| Study | Domain | Judgement | Supporting information |
| --- | --- | --- | --- |
| Deng 2022 | Random sequence generation (selection bias) | Unclear risk | The report states that 160 patients were randomized 1:1:1:1; the sequence-generation method is not described in the accessible record. |
| Deng 2022 | Allocation concealment (selection bias) | Unclear risk | No usable allocation-concealment method was identified. |
| Deng 2022 | Blinding of participants and personnel (performance bias) | High risk | Music and aroma exposure were perceptible and convincing participant/personnel masking was not reported. |
| Deng 2022 | Blinding of outcome assessment (detection bias) | High risk | Pain and anxiety were measured by participant-rated visual analogue scales; awareness of treatment could influence these subjective outcomes. |
| Deng 2022 | Incomplete outcome data (attrition bias) | Low risk | All 160 randomized participants and four equal groups are reported; no differential missing outcome data are apparent in the accessible results. |
| Deng 2022 | Selective reporting (reporting bias) | Unclear risk | No prospectively accessible protocol/registration was verified for comparison with the published outcomes. |
| Deng 2022 | Other bias | Unclear risk | Limited access to the complete methods prevents exclusion of additional design or conduct concerns. |
| Sayadi 2024 | Random sequence generation (selection bias) | Unclear risk | The study is described as randomized, but an adequate sequence-generation procedure was not available for verification. |
| Sayadi 2024 | Allocation concealment (selection bias) | Unclear risk | Allocation concealment was not available for verification. |
| Sayadi 2024 | Blinding of participants and personnel (performance bias) | High risk | Rosemary aroma and music are readily distinguishable; participant/personnel masking was not established. |
| Sayadi 2024 | Blinding of outcome assessment (detection bias) | High risk | The principal anxiety outcome was subjective and could be influenced by knowledge of intervention. |
| Sayadi 2024 | Incomplete outcome data (attrition bias) | Unclear risk | The accessible record does not give sufficient randomized, excluded, and analyzed numbers with reasons. |
| Sayadi 2024 | Selective reporting (reporting bias) | Unclear risk | No prospective protocol or registration was verified. |
| Sayadi 2024 | Other bias | Unclear risk | The limited methods record does not permit a reliable judgement on other bias. |
| Law 2023 | Random sequence generation (selection bias) | Unclear risk | Block randomization to three groups is reported, but the method used to generate the block sequence is not specified. |
| Law 2023 | Allocation concealment (selection bias) | Unclear risk | No adequate concealment mechanism was identified in the accessible report. |
| Law 2023 | Blinding of participants and personnel (performance bias) | High risk | Participants could distinguish aroma and music from control conditions. |
| Law 2023 | Blinding of outcome assessment (detection bias) | High risk | Pain VAS and STAI anxiety were participant reported and therefore vulnerable to unblinded assessment. |
| Law 2023 | Incomplete outcome data (attrition bias) | Low risk | Ninety participants were randomized and results are reported for 30 per group. |
| Law 2023 | Selective reporting (reporting bias) | Unclear risk | No prospective protocol or registration was verified. |
| Law 2023 | Other bias | Low risk | No additional material baseline or conduct concern was identified in the accessible report. |
| Rezaei 2024 | Random sequence generation (selection bias) | Low risk | A six-block randomization schedule with all six ABC permutations was reported. |
| Rezaei 2024 | Allocation concealment (selection bias) | Low risk | Blocks were concealed in matte envelopes and participants selected their allocation envelope. |
| Rezaei 2024 | Blinding of participants and personnel (performance bias) | High risk | Participants and the intervention team could distinguish lavender, music, and placebo conditions. |
| Rezaei 2024 | Blinding of outcome assessment (detection bias) | High risk | Although the research assistant was blinded, the primary STAI anxiety outcome was self-reported by unblinded participants. |
| Rezaei 2024 | Incomplete outcome data (attrition bias) | Low risk | All 195 randomized participants were analyzed; none was excluded after randomization. |
| Rezaei 2024 | Selective reporting (reporting bias) | Low risk | The trial was registered prospectively (IRCT20090810002324N17, 22 Dec 2020) and the reported primary/secondary outcomes correspond to the prespecified trial description. |
| Rezaei 2024 | Other bias | Low risk | Baseline anxiety differed, but the authors adjusted the analysis; no additional material bias was identified. |
| Rohi Ganji 2022 | Random sequence generation (selection bias) | Low risk | A blocked sequence was generated using sealedenvelope.com, with blocks of eight. |
| Rohi Ganji 2022 | Allocation concealment (selection bias) | Low risk | Group assignments were placed in opaque envelopes/cards before enrolment. |
| Rohi Ganji 2022 | Blinding of participants and personnel (performance bias) | High risk | Music and aroma exposure could not be masked from participants or administering staff. |
| Rohi Ganji 2022 | Blinding of outcome assessment (detection bias) | High risk | The data collector was blinded, but anxiety was participant-reported and could be influenced by treatment awareness. |
| Rohi Ganji 2022 | Incomplete outcome data (attrition bias) | Low risk | Randomized and analyzed numbers were adequately reported without important differential loss. |
| Rohi Ganji 2022 | Selective reporting (reporting bias) | Unclear risk | No sufficiently detailed prospective protocol comparison was available for this assessment. |
| Rohi Ganji 2022 | Other bias | Low risk | No additional material source of bias was identified. |
| Zamanifar 2020 | Random sequence generation (selection bias) | Low risk | Within each ward stratum, numbered cards 1–4 were drawn without replacement to allocate nurses to four groups. |
| Zamanifar 2020 | Allocation concealment (selection bias) | Unclear risk | The report does not describe a mechanism that prevented foreknowledge of the next assignment. |
| Zamanifar 2020 | Blinding of participants and personnel (performance bias) | High risk | The principal researcher and nurses knew the intervention received. |
| Zamanifar 2020 | Blinding of outcome assessment (detection bias) | High risk | A blinded data collector/statistician is reported, but Beck anxiety was self-reported by unblinded nurses. |
| Zamanifar 2020 | Incomplete outcome data (attrition bias) | Low risk | No exclusions or losses after allocation were reported; 120 nurses were analyzed. |
| Zamanifar 2020 | Selective reporting (reporting bias) | Unclear risk | No prospective protocol or trial registry was available for outcome comparison. |
| Zamanifar 2020 | Other bias | Unclear risk | Purposeful recruitment and limited reporting leave residual concerns, although these do not prove a specific additional bias. |
| Wen 2023 | Random sequence generation (selection bias) | Unclear risk | Random allocation 1:1:1:1 is reported, but the sequence-generation procedure was not available for verification. |
| Wen 2023 | Allocation concealment (selection bias) | Unclear risk | No adequate concealment procedure was available for verification. |
| Wen 2023 | Blinding of participants and personnel (performance bias) | High risk | Music and lavender exposure are perceptible despite the report's 'double-blind' label. |
| Wen 2023 | Blinding of outcome assessment (detection bias) | High risk | Primary STAI-1 and SAS anxiety outcomes were self-reported by participants aware of the intervention. |
| Wen 2023 | Incomplete outcome data (attrition bias) | Low risk | Two hundred participants were reported with 50 in each randomized group. |
| Wen 2023 | Selective reporting (reporting bias) | Unclear risk | No prospectively accessible protocol/registration was verified. |
| Wen 2023 | Other bias | Low risk | No other material bias was apparent from the accessible report. |
| Janthasila 2023 | Random sequence generation (selection bias) | Unclear risk | The trial is randomized, but the exact sequence-generation method is not reported clearly enough for a low-risk judgement. |
| Janthasila 2023 | Allocation concealment (selection bias) | Unclear risk | Allocation concealment is not adequately described. |
| Janthasila 2023 | Blinding of participants and personnel (performance bias) | High risk | The authors explicitly state that participants and dentists were not blinded. |
| Janthasila 2023 | Blinding of outcome assessment (detection bias) | High risk | The authors state that assessors were not blinded, and anxiety/fear outcomes were susceptible to knowledge of allocation. |
| Janthasila 2023 | Incomplete outcome data (attrition bias) | Low risk | The analyzed sample and group counts are reported without important unexplained differential loss. |
| Janthasila 2023 | Selective reporting (reporting bias) | Unclear risk | No prospective protocol or registration was verified. |
| Janthasila 2023 | Other bias | Low risk | No additional material bias was identified. |
| Yu 2022 | Random sequence generation (selection bias) | Unclear risk | Random allocation could not be verified from an accessible full report. |
| Yu 2022 | Allocation concealment (selection bias) | Unclear risk | Allocation concealment could not be verified. |
| Yu 2022 | Blinding of participants and personnel (performance bias) | High risk | Aromatherapy plus five-element music is perceptible and masking was not established. |
| Yu 2022 | Blinding of outcome assessment (detection bias) | High risk | Pain, anxiety, and depression are subjective outcomes; assessor/participant masking was not established. |
| Yu 2022 | Incomplete outcome data (attrition bias) | Unclear risk | Insufficient participant-flow information was available. |
| Yu 2022 | Selective reporting (reporting bias) | Unclear risk | No protocol or registry was verified. |
| Yu 2022 | Other bias | Unclear risk | The study design and baseline comparability could not be assessed from the accessible record. |
| Hohneck 2025 | Random sequence generation (selection bias) | Unclear risk | Randomization 1:1 is reported, but an adequate sequence-generation method was not available for verification. |
| Hohneck 2025 | Allocation concealment (selection bias) | Unclear risk | Allocation concealment was not available for verification. |
| Hohneck 2025 | Blinding of participants and personnel (performance bias) | High risk | Participants could distinguish sound alone from sound plus aromatherapy. |
| Hohneck 2025 | Blinding of outcome assessment (detection bias) | High risk | Psychophysiological/self-reported outcomes may be influenced by treatment awareness; assessor masking was not established for those outcomes. |
| Hohneck 2025 | Incomplete outcome data (attrition bias) | Unclear risk | The accessible report did not provide sufficient flow detail for a firm attrition judgement. |
| Hohneck 2025 | Selective reporting (reporting bias) | Unclear risk | No prospective protocol comparison was verified. |
| Hohneck 2025 | Other bias | Unclear risk | The exploratory design and limited accessible methods leave other bias unclear. |
| Abdalhai 2024 | Random sequence generation (selection bias) | Low risk | Simple randomization was generated at random.org for 56 children. |
| Abdalhai 2024 | Allocation concealment (selection bias) | Unclear risk | The report does not describe who concealed the sequence or how assignments were protected before enrolment. |
| Abdalhai 2024 | Blinding of participants and personnel (performance bias) | High risk | Children and treating personnel could distinguish music and aroma from the empty-mask control. |
| Abdalhai 2024 | Blinding of outcome assessment (detection bias) | High risk | The primary FIS anxiety outcome was self-reported by unblinded children; blinded video raters applied only to FLACC pain. |
| Abdalhai 2024 | Incomplete outcome data (attrition bias) | Low risk | All 56 randomized children (28 per group) were included in the reported results. |
| Abdalhai 2024 | Selective reporting (reporting bias) | Unclear risk | Registration NCT05759286 occurred after recruitment had begun, so prospective outcome specification was not established. |
| Abdalhai 2024 | Other bias | Low risk | No additional material bias was identified from the full text. |
| Akbaş Uysal 2024 | Random sequence generation (selection bias) | Unclear risk | The study is described as randomized, but the sequence-generation method was not verified. |
| Akbaş Uysal 2024 | Allocation concealment (selection bias) | Unclear risk | No adequate concealment method was verified. |
| Akbaş Uysal 2024 | Blinding of participants and personnel (performance bias) | High risk | Aromatherapy and music exposure were perceptible. |
| Akbaş Uysal 2024 | Blinding of outcome assessment (detection bias) | High risk | Pain, anxiety, and stress were subjective participant-reported outcomes. |
| Akbaş Uysal 2024 | Incomplete outcome data (attrition bias) | Unclear risk | The accessible record did not provide enough participant-flow detail for a firm judgement. |
| Akbaş Uysal 2024 | Selective reporting (reporting bias) | Unclear risk | The ClinicalTrials.gov record (NCT06024954) was posted around/after publication rather than prospectively; selective reporting cannot be excluded but is not demonstrated. |
| Akbaş Uysal 2024 | Other bias | Unclear risk | The late registration and limited accessible methods leave other bias unclear. |
| Mousavi 2025 | Random sequence generation (selection bias) | Low risk | A permuted-block method assigned 72 participants equally to four groups. |
| Mousavi 2025 | Allocation concealment (selection bias) | Low risk | Sequentially numbered sealed envelopes were used; recruitment/randomization were overseen separately. |
| Mousavi 2025 | Blinding of participants and personnel (performance bias) | High risk | Music and aroma could be recognized by participants and treating endodontists. |
| Mousavi 2025 | Blinding of outcome assessment (detection bias) | Low risk | Blood pressure and heart rate were objectively recorded with an electronic device by a blinded examiner. |
| Mousavi 2025 | Incomplete outcome data (attrition bias) | Low risk | All 72 randomized participants completed the study and were analyzed. |
| Mousavi 2025 | Selective reporting (reporting bias) | Low risk | The prospectively registered IRCT record (IRCT20191228045910N1) and publication report the specified physiological outcomes. |
| Mousavi 2025 | Other bias | Low risk | Baseline characteristics were comparable and no additional material concern was identified. |
| Bolandi 2025 | Random sequence generation (selection bias) | Low risk | Group assignment used equal cards/chits prepared for the four study arms. |
| Bolandi 2025 | Allocation concealment (selection bias) | Low risk | Assignments were placed in opaque envelopes before selection. |
| Bolandi 2025 | Blinding of participants and personnel (performance bias) | High risk | Music could not be masked; placebo headphones/aroma did not fully blind all intervention contrasts. |
| Bolandi 2025 | Blinding of outcome assessment (detection bias) | Low risk | A trained outcome assessor blinded to allocation measured objective physiological indices. |
| Bolandi 2025 | Incomplete outcome data (attrition bias) | Low risk | The randomized and analyzed sample was adequately accounted for without important differential loss. |
| Bolandi 2025 | Selective reporting (reporting bias) | Unclear risk | No sufficiently detailed prospective protocol comparison was verified. |
| Bolandi 2025 | Other bias | Low risk | No additional material baseline or conduct concern was identified. |
| Gür 2026 | Random sequence generation (selection bias) | Low risk | Random.org generated a balanced random integer sequence for four groups. |
| Gür 2026 | Allocation concealment (selection bias) | Low risk | An independent researcher used sealed opaque envelopes to implement allocation. |
| Gür 2026 | Blinding of participants and personnel (performance bias) | High risk | Participants and the primary investigator were not blinded to the sensory interventions. |
| Gür 2026 | Blinding of outcome assessment (detection bias) | High risk | VAS pain/anxiety/fear and SAI were participant-reported outcomes; blinded statistical analysis does not remove outcome-measurement bias. |
| Gür 2026 | Incomplete outcome data (attrition bias) | Low risk | All 128 assigned participants were included in the reported analyses. |
| Gür 2026 | Selective reporting (reporting bias) | Low risk | The trial was registered as NCT05622383 and the reported outcomes correspond to the registered trial description. |
| Gür 2026 | Other bias | Low risk | No additional material source of bias was identified. |
| Öner 2026 | Random sequence generation (selection bias) | Low risk | Participant numbers were assigned to three groups using a computer-aided random allocation procedure. |
| Öner 2026 | Allocation concealment (selection bias) | Unclear risk | No adequate concealment mechanism was identified in the accessible report. |
| Öner 2026 | Blinding of participants and personnel (performance bias) | High risk | Students could recognize aromatherapy and music during injection practice. |
| Öner 2026 | Blinding of outcome assessment (detection bias) | High risk | Anxiety/stress/self-esteem outcomes were participant reported despite the single-blind label. |
| Öner 2026 | Incomplete outcome data (attrition bias) | Low risk | The 102 enrolled participants are reported as 34 per group through analysis. |
| Öner 2026 | Selective reporting (reporting bias) | Unclear risk | No prospective protocol or registration was verified. |
| Öner 2026 | Other bias | Low risk | No additional material baseline or conduct concern was identified. |
| Kovvali 2025 | Random sequence generation (selection bias) | Unclear risk | The report says 54 participants were randomly assigned, but gives no reproducible sequence-generation method. |
| Kovvali 2025 | Allocation concealment (selection bias) | Unclear risk | Allocation concealment is not reported. |
| Kovvali 2025 | Blinding of participants and personnel (performance bias) | High risk | Participants knew whether they received combined therapy, music alone, or control. |
| Kovvali 2025 | Blinding of outcome assessment (detection bias) | High risk | Anxiety, sleep, and affect outcomes were self-reported by unblinded participants. |
| Kovvali 2025 | Incomplete outcome data (attrition bias) | Low risk | All 54 eligible participants were allocated 18 per group and reported in the analysis. |
| Kovvali 2025 | Selective reporting (reporting bias) | Unclear risk | No protocol or registry was reported. |
| Kovvali 2025 | Other bias | High risk | Internal statistical inconsistencies (for example F=0.821 reported with p=0.017) and sparse analytic detail raise a material reporting/analysis concern. |
| Bagaria 2026 | Random sequence generation (selection bias) | Low risk | Participants were allocated by chit picking, an unpredictable lottery-type method when implemented as described. |
| Bagaria 2026 | Allocation concealment (selection bias) | Low risk | The report states that allocation was obscured through chit picking before assignment. |
| Bagaria 2026 | Blinding of participants and personnel (performance bias) | High risk | The sensory/distraction conditions were apparent to children and clinicians. |
| Bagaria 2026 | Blinding of outcome assessment (detection bias) | High risk | A blinded observer assessed procedural behavior/pain, but the anxiety measure remained susceptible to participant awareness; the judgement is anchored to anxiety. |
| Bagaria 2026 | Incomplete outcome data (attrition bias) | Low risk | The enrolled/analyzed groups were reported without important differential loss. |
| Bagaria 2026 | Selective reporting (reporting bias) | Unclear risk | No prospective protocol or registration was verified. |
| Bagaria 2026 | Other bias | Low risk | No additional material source of bias was identified. |
| Sakee 2026 | Random sequence generation (selection bias) | Low risk | The report describes simple random allocation using 60 differently numbered items/cards. |
| Sakee 2026 | Allocation concealment (selection bias) | Unclear risk | No adequate mechanism preventing foreknowledge of assignment was established. |
| Sakee 2026 | Blinding of participants and personnel (performance bias) | High risk | Lavender/rosemary with music and the behavioral control were distinguishable. |
| Sakee 2026 | Blinding of outcome assessment (detection bias) | High risk | Behavioral/anxiety assessment was not shown to be performed by an assessor blinded to group allocation. |
| Sakee 2026 | Incomplete outcome data (attrition bias) | Low risk | The 60 randomized children were reported across the study groups without important unexplained loss. |
| Sakee 2026 | Selective reporting (reporting bias) | Unclear risk | No prospective protocol or registry was verified. |
| Sakee 2026 | Other bias | Unclear risk | The accessible report does not provide enough information to exclude other design or analysis concerns. |
| Patil 2020 | Random sequence generation (selection bias) | Unclear risk | The accessible citation does not provide a reproducible random sequence-generation method. |
| Patil 2020 | Allocation concealment (selection bias) | Unclear risk | Allocation concealment could not be verified. |
| Patil 2020 | Blinding of participants and personnel (performance bias) | High risk | VR, audio-analgesia, and white noise plus aromatherapy were readily distinguishable. |
| Patil 2020 | Blinding of outcome assessment (detection bias) | High risk | Anxiety/disruptive behavior outcomes could be influenced by unblinded participants and observers; assessor blinding was not verified. |
| Patil 2020 | Incomplete outcome data (attrition bias) | Unclear risk | Randomized, excluded, and analyzed numbers with reasons were not sufficiently available. |
| Patil 2020 | Selective reporting (reporting bias) | Unclear risk | No prospective protocol or registration was verified. |
| Patil 2020 | Other bias | Unclear risk | The full design, baseline balance, and analysis could not be assessed. |
| Son 2019 | Random sequence generation (selection bias) | Low risk | An independent research assistant generated assignments using the Research Randomizer. |
| Son 2019 | Allocation concealment (selection bias) | Unclear risk | The publication does not clearly describe concealment of the sequence until enrolment. |
| Son 2019 | Blinding of participants and personnel (performance bias) | High risk | Students could recognize aroma and music, so the 'double-blind' label does not establish participant masking. |
| Son 2019 | Blinding of outcome assessment (detection bias) | High risk | Test/state anxiety and stress were self-reported by participants aware of their intervention; skill assessment has a different bias profile. |
| Son 2019 | Incomplete outcome data (attrition bias) | Low risk | Ninety-eight participants were included in the final analysis with group numbers reported. |
| Son 2019 | Selective reporting (reporting bias) | Unclear risk | No prospective protocol or registry was verified. |
| Son 2019 | Other bias | Low risk | No additional material baseline or conduct concern was identified. |

# Supplementary Table S3. JBI appraisal of controlled nonrandomized/quasi-experimental studies (3 controlled nonrandomized or quasi-experimental studies)

Yang et al. (2025), Mahendra et al. (2020), and Masmirawati et al. (2025) were assessed using the JBI checklist for quasi-experimental studies. Mahendra et al. was placed in this table because the report described an experimental four-group study but did not report a verifiable random-allocation procedure.

| Study | JBI item | Question | Judgement | Supporting rationale |
| --- | --- | --- | --- | --- |
| Yang 2025 | Q1 | Is it clear what is the cause and what is the effect? | Yes | Intervention exposure preceded the post-intervention outcome assessment. |
| Yang 2025 | Q2 | Were the participants included in comparisons similar? | Unclear | The accessible report did not provide enough baseline detail to confirm comparability across all relevant characteristics. |
| Yang 2025 | Q3 | Apart from the intervention, did participants receive similar care? | Yes | The comparison groups received similar procedures apart from the addition of music. |
| Yang 2025 | Q4 | Was there a control group? | Yes | An aromatherapy-only concurrent comparison group was used. |
| Yang 2025 | Q5 | Were there multiple measurements before and after the intervention? | No | The report used a pretest/post-test structure but did not describe multiple measurements on both sides of the intervention. |
| Yang 2025 | Q6 | Was follow-up complete, or were differences in follow-up adequately described and analyzed? | Unclear | Complete follow-up and handling of missing outcome data were not sufficiently detailed in the accessible report. |
| Yang 2025 | Q7 | Were outcomes measured in the same way for the groups? | Yes | The same anxiety, stress, depression, and physiological measures were used across groups. |
| Yang 2025 | Q8 | Were outcomes measured reliably? | Yes | Validated self-report instruments and standardized physiological measurements were reported. |
| Yang 2025 | Q9 | Was appropriate statistical analysis used? | Unclear | The quasi-experimental design and available reporting did not permit full verification of model assumptions and confounding control. |
| Mahendra 2020 | Q1 | Is it clear what is the cause and what is the effect? | Yes | The sensory intervention was delivered before the post-treatment anxiety and physiological measurements. |
| Mahendra 2020 | Q2 | Were the participants included in comparisons similar? | Unclear | Group allocation and baseline comparability were not described in sufficient detail. |
| Mahendra 2020 | Q3 | Apart from the intervention, did participants receive similar care? | Yes | The reported groups differed principally in the assigned sensory intervention. |
| Mahendra 2020 | Q4 | Was there a control group? | Yes | Concurrent comparison groups were included. |
| Mahendra 2020 | Q5 | Were there multiple measurements before and after the intervention? | No | Multiple repeated measurements before and after treatment were not reported. |
| Mahendra 2020 | Q6 | Was follow-up complete, or were differences in follow-up adequately described and analyzed? | Unclear | Participant flow and losses after allocation were insufficiently reported. |
| Mahendra 2020 | Q7 | Were outcomes measured in the same way for the groups? | Yes | Outcomes were assessed using the same procedures across groups. |
| Mahendra 2020 | Q8 | Were outcomes measured reliably? | Unclear | The accessible report did not provide enough information about assessor training or reliability. |
| Mahendra 2020 | Q9 | Was appropriate statistical analysis used? | Unclear | Statistical methods were reported incompletely and could not be fully verified. |
| Masmirawati 2025 | Q1 | Is it clear what is the cause and what is the effect? | Yes | The intervention preceded the post-intervention anxiety assessment. |
| Masmirawati 2025 | Q2 | Were the participants included in comparisons similar? | Unclear | The report did not provide enough baseline information to establish complete group comparability. |
| Masmirawati 2025 | Q3 | Apart from the intervention, did participants receive similar care? | Yes | The concurrent groups were reported to receive similar care apart from the sensory intervention. |
| Masmirawati 2025 | Q4 | Was there a control group? | Yes | A concurrent comparison group was included. |
| Masmirawati 2025 | Q5 | Were there multiple measurements before and after the intervention? | No | The study used pretest and post-test measurements rather than multiple measurements before and after treatment. |
| Masmirawati 2025 | Q6 | Was follow-up complete, or were differences in follow-up adequately described and analyzed? | Yes | The analyzed sample and completion of outcome measurement were reported without important differential loss. |
| Masmirawati 2025 | Q7 | Were outcomes measured in the same way for the groups? | Yes | The same anxiety measure and timing were applied to the groups. |
| Masmirawati 2025 | Q8 | Were outcomes measured reliably? | Unclear | The accessible report did not provide sufficient detail on assessor reliability or masking. |
| Masmirawati 2025 | Q9 | Was appropriate statistical analysis used? | Yes | The reported analysis was appropriate for the stated pretest/post-test group comparisons. |

# Supplementary Figure S1. Distribution of RoB 1 judgements by domain

Figure S1. Distribution of Low risk, Unclear risk, and High risk judgements across the 21 randomized studies. Bar lengths represent the percentage distribution within each RoB 1 domain.

# Supplementary Figure S2. JBI appraisal summary

Figure S2. JBI appraisal for Yang et al. (2025), Mahendra et al. (2020), and Masmirawati et al. (2025). Green (+), Yes; yellow (?), Unclear; red (−), No. Q1-Q9 correspond to the questions listed in Supplementary Table S4.
